# Highly Sensitive and Selective Surface Acoustic Wave Ammonia Sensor Operated at Room Temperature with a Polyacrylic Acid Sensing Layer

**DOI:** 10.3390/s22176349

**Published:** 2022-08-24

**Authors:** Weiqiang Wang, Yuanjun Guo, Wenkai Xiong, Yongqing Fu, Ahmed Elmarakbi, Xiaotao Zu

**Affiliations:** 1 School of Physics, University of Electronic Science and Technology of China, Chengdu 610054, China; 2 Faculty of Engineering & Environment, University of Northumbria, Newcastle upon Tyne, NE1 8ST, UK

**Keywords:** surface acoustic wave (SAW), polyacrylic acid (PAA), ammonia sensor

## Abstract

In this study, polyacrylic acid (PAA) films were deposited onto a quartz surface acoustic wave (SAW) resonator using a spin-coating technique for ammonia sensing operated at room temperature, and the sensing mechanisms and performance were systematically studied. The oxygen-containing functional groups on the surfaces of the PAA film make it sensitive and selective to ammonia molecules, even when tested at room temperature. The ammonia molecules adsorbed by the oxygen-containing functional groups of PAA (e.g., hydroxyl and epoxy groups) increase the membrane’s stiffness, which was identified as the primary mechanism leading to the positive frequency shifts. However, mass loading due to adsorption of ammonia molecules is not a major reason as it will result in a negative frequency shifts. When the PAA coated SAW sensor was exposed to ammonia with a low concentration of 500 ppb, it showed a positive frequency shift of 225 Hz, with both good repeatability and stability, as well as a good selectivity to ammonia compared with those to C_2_H_5_OH, H_2_, HCl, H_2_S, CO, NO_2_, NO, and CH_3_COCH_3_.

## 1. Introduction

Ammonia is one of the most common toxic gases and is widely used in medical, chemical, gas, and food industries, and thus the risk of ammonia leakage or contamination is very high in our daily life. Excessive ammonia inhalation can lead to respiratory problems, severe headaches, sore throats, loss of smell and chest pain. Due to these potential risks, development of highly sensitive and selective ammonia sensors is of great importance [1,2,3]. At present, various types of ammonia sensors have been developed, including metal oxide semiconductor sensors, electrochemical sensors, and surface acoustic wave (SAW) sensors [4,5,6,7], etc. 

In recent years, SAW-based ammonia sensors have attracted extensive attention [8,9,10,11]. They have the advantages including high sensitivity, high speed, good reliability, high precision, and low cost [12,13,14]. A typical SAW-based gas sensor consists of a resonator with a specific sensing film and its corresponding oscillator [15,16,17]. The core part is the SAW resonator [18], which is normally coated with a sensitive film, and the SAW device’s resonate frequency is changed after adsorption or chemical combination of ammonia molecules [19,20]. Changes of conductivity of the sensitive film (i.e., electrical loading), effective mass of the film (i.e., mass loading), and/or modulus of elasticity (i.e., elastic loading) can change the resonant frequency of the SAW devices [7,16,21]. 

So far, many materials have been used as sensing layers to detect ammonia gas, such as indium oxide, titanium oxide, cerium oxide, carbon nanotubes, and graphene [17,22,23,24,25,26,27,28,29]. For example, Tang et al. [10] deposited ZnO/SiO_2_ composite films onto the surface of SAW devices using a sol–gel method, and obtained a limit of ammonia detection of 5 ppm. Guo et al. [27] prepared SiO_2_-SnO_2_ sensitive thin films onto the SAW device using sol–gel and spin-coating methods and obtained a limit of ammonia detection of 3 ppm. Hung et al. [29] deposited reduced graphene oxide/poly (diketopyrrolopyrrolethiophene-thieno [3,2-b]thiophene-thiophene) (rGO/DPP2T-TT) onto the SAW device using a dripping method and obtained a limit of ammonia detection of 0.5 ppm. Clearly, it is crucial to find a material with a low cost and a simple method to form a sensitive film for SAW-based ammonia sensors. Polyacrylic acid (PAA) polymer, a cheap organic soft matter, has a large number of carboxyl groups on its surface and forms carboxyl and ammonia ions when it contacts ammonia molecules. These make it a good adsorbent for toxic gases such as ammonia [30] and thus a suitable sensitive film for ammonia. Therefore, we believe that an integration of this sensing layer onto the SAW device will achieve an ammonia sensor with a high sensitivity. However, as far as we have searched, this has never been reported for SAW-based ammonia sensors. 

In this work, a PAA-based ammonia SAW sensor was made on an ST cut quartz substrate, and its ammonia-sensing mechanism operated at room temperature was studied. Results proved that the PAA SAW sensor has both good sensitivity and selectivity to ammonia when operated at room temperature.

## 2. Materials and Methods

### 2.1. SAW Resonator and Sensing Layer

**Figure 1:**
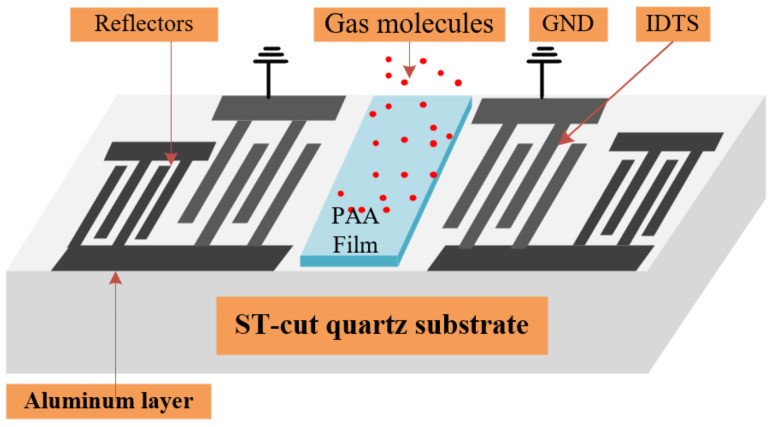
Schematic illustration of the SAW resonator.

Two interdigital transducers (IDTs), each side with 30 finger pairs and reflection gratings of 100 pairs, were used to construct the SAW resonators on ST-cut quartz substrate. Using a traditional photolithography and lift-off method, the IDTs and reflection gratings were prepared with a 200 nm thick aluminum, a period of 16 μm, and an aperture of 3 mm. The resonator’s intrinsic frequency was designed to be 200 MHz. The configuration of the SAW resonator is schematically depicted in Figure 1.

PAA solutions with concentrations of 0.01 M, 0.05 M, 0.1 M, and 0.2 M (bought from Kelong Chemical Reagent Factory, Chengdu, China) were synthesized in a container with a magnetic stirrer at room temperature for 90 min. They were then ultrasonically agitated for two hours after being aged for 24 h. In order to prepare a uniform PAA film, the produced PAA solutions were spin-coated onto ST-cut quartz resonators at a speed of 7000 rpm for 30 s and then dried at 60 °C for 8 min. Finally, the bonded aluminum wire was used to join the SAW resonator to the external circuit and control system to form the whole SAW sensor.

### 2.2. Sensing and Characterization Platform

**Figure 2:**
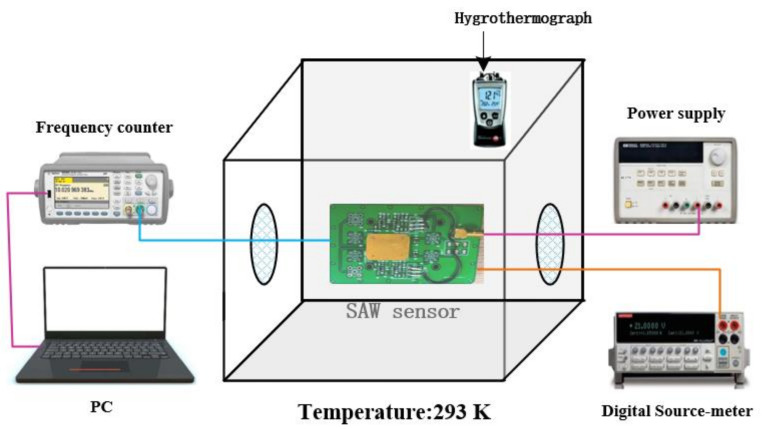
SAW gas sensing test system.

Figure 2 shows the gas sensing system, which mainly includes a 20 L closed chamber, a DC power supply (Agilent, E3631A), a hydro thermometer, a digital source meter (Keithley 2400), and a frequency counter (Agilent 53210A). Commercially standard gases of NH_3_ (2 vol%), H_2_S (2 vol%), H_2_ (2 vol%), CO (2 vol%), NO_2_ (2 vol%), CH_3_COCH_3_ (2 vol%), HCl (2 vol%), and C_2_H_5_OH (2 vol%) in the dry air were purchased from the China Academy of Measurement and Testing Technology. During the sensing process, the testing environment was controlled at room temperature of 20 °C and 30% RH with the precise control of the dried air and humidifier. The sensor was put in the 20 L closed chamber. After the sensor reached a steady state of frequency, a precisely controlled syringe was used to fill the test chamber with the given amount of ammonia. By controlling the volume of ammonia injected into the chamber, different concentrations of ammonia were obtained. For example, in an environment with 30% RH, injection of 20 mL of standard ammonia within the 20 L air achieved 20 ppm of ammonia. 

A field emission scanning electron microscope (FE-SEM, FEI Inspect F50), a Fourier transform infrared spectroscope (FTIR, Nicolet IS 10, Thermo Fisher Scientific, Waltham, MA, USA), and a digital source meter (Keithley 2400) were used to characterize the morphologies, infrared absorption spectra of the prepared PAA films, and the changes in the conductance of the sensor before and after it was exposed to ammonia, respectively.

## 3. Results and Discussion

### 3.1. Characterization of Sensing Film

**Figure 3:**
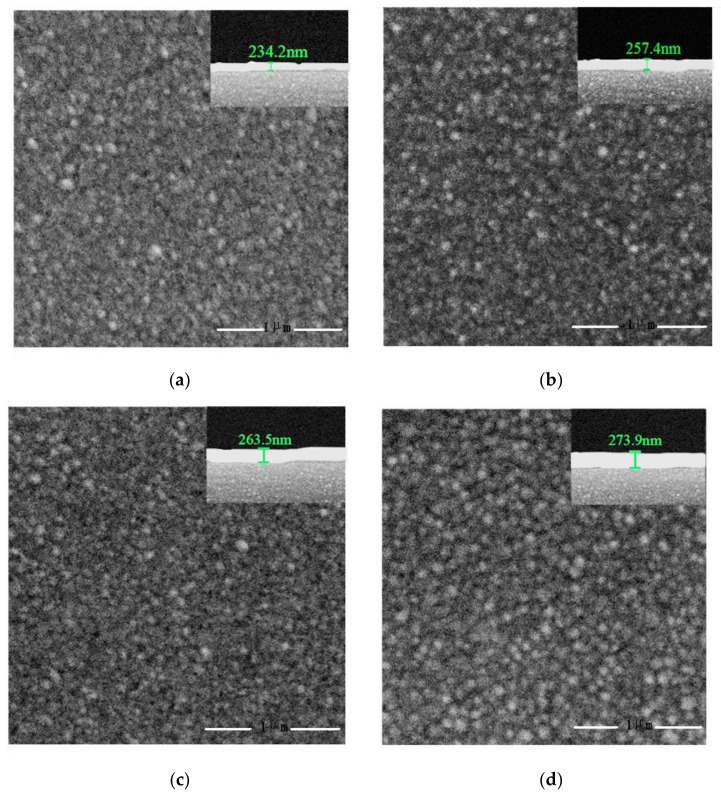
SEM images of surface and cross-section morphologies (inset) of PAA films with different concentrations: (**a**) 0.01 M; (**b**) 0.05 M; (**c**) 0.10 M; (**d**) 0.20 M.

Figure 3 shows SEM images of surface and cross-section morphologies of PAA films with different concentrations. It can be seen that the prepared PAA films were quite dense, and when the concentration of PAA increased from 0.01 M to 0.2 M, the corresponding film’s thickness increased from 234.2 nm to 273.9 nm.

**Figure 4:**
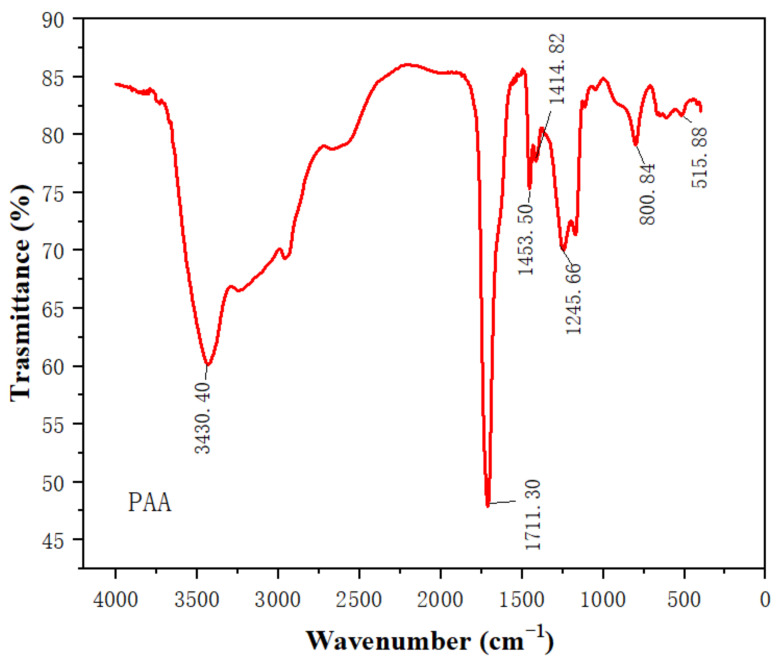
FTIR spectra of PAA film.

FTIR analysis results of PAA are shown in Figure 4. The band at 3430.16 cm^−1^ is due to the O-H stretching mode and the presence of hydrogen bonding [31]. The strong band at 1711.30 cm^−1^ is due to the asymmetric stretching of –COO– [32,33]. The bands at 1453.50 and 1414.82 cm^−1^ reveal the symmetric stretching modes of –COO– [34]. FTIR results clearly indicate that there are various hydroxyl groups and carboxyl groups on the surface of PAA film. The carboxyl groups on the PAA film were reported to have large adsorption energy for ammonia molecules [30].

### 3.2. Gas Sensing Results and Sensing Mechanisms

**Figure 5:**
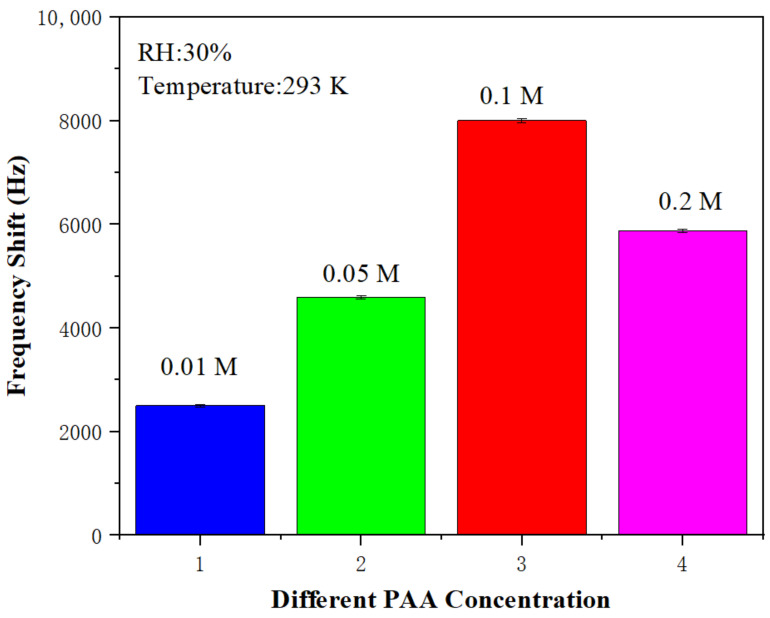
Responses of the SAW ammonia sensor prepared by PAA with different mole ratios to 20 ppm ammonia.

Figure 5 shows the frequency shifts (or the responses) of SAW sensors with sensitive films of different PAA molar ratios when exposed to 20 ppm ammonia at 293 K and 30% RH. It can be seen from Figure 5 that the sensor made of 0.1 M PAA solution had the largest response of 8000 Hz. Therefore, the sensor prepared with 0.1 M PAA solution was selected for the subsequent performance tests.

**Figure 6:**
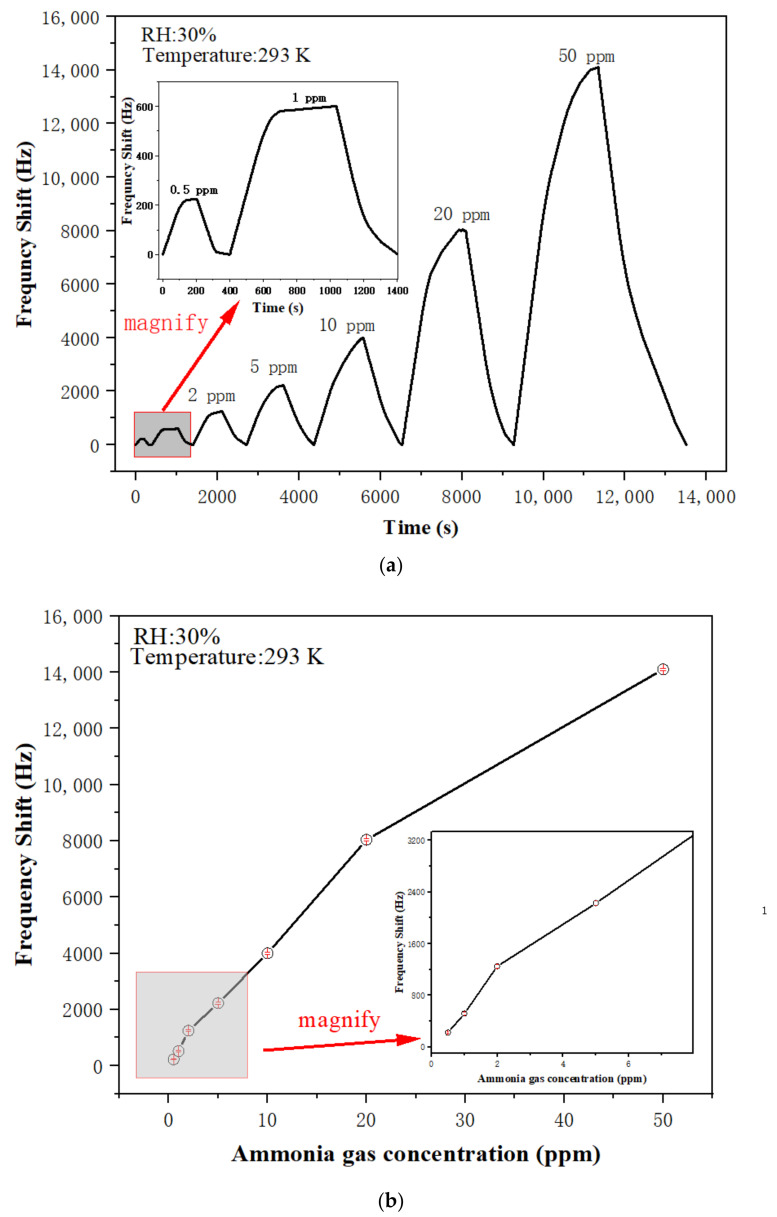
(**a**) Dynamic responses of PAASAW sensors to ammonia gases with different concentrations; (**b**) the relationship between frequency shift and ammonia gas concentrations.

Figure 6a shows the frequency shifts of PAA SAW sensor coated with 0.1 M PAA solution to different concentrations of ammonia between 500 ppb to 50 ppm. As can be seen from Figure 6a, the PAA-coated SAW sensor achieved good limits of detection (LOD) and large frequency shifts when exposed to ammonia. It had a frequency shift response of 225 Hz to 0.5 ppm ammonia. Figure 6b shows the relationship between frequency shift and ammonia gas concentration. The obtained slopes of the responses for the PAA SAW sensor in the range of 0.5–2 ppm and 2–20 ppm were 750 and 400 Hz/ppm, respectively. Table 1 summarizes the LODs of SAW ammonia sensors with various sensitive layers, reported in literature, as well as that obtained from this paper. Clearly, the SAW sensor with the PAA layer in this study showed one of the best LODs for the ammonia sensing compared with the others [7,10,17,21,26,27,28,29]. Meanwhile, the SAW sensor with the PAA layer also showed good repeatability and stability, as well as a good selectivity to ammonia, if compared with those to various gases of C_2_H_5_OH, H_2_, HCl, H_2_S, CO, NO_2_, NO, and CH_3_COCH_3_ (seen in Section 3.3). 

**Figure 7:**
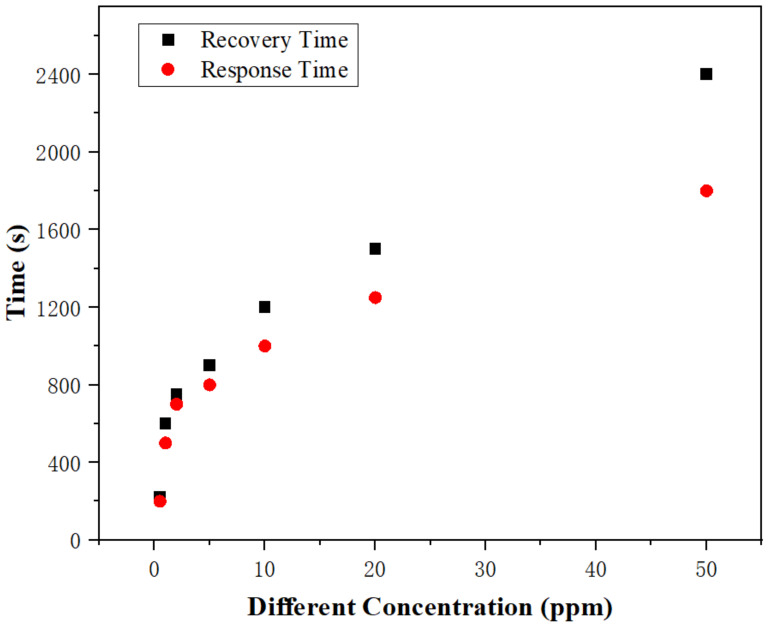
Response and recovery times of the PAA SAW ammonia sensor at different concentrations of ammonia.

When exposed to ammonia gases with various concentrations, the SAW sensor’s response and recovery times were quite different, which are shown in Figure 7. Here, the response time was defined as the time needed for the sensor’s frequency shift to reach 90% of its total frequency shift after being exposed to ammonia. Similar to this, the recovery time was defined as the time needed for the sensor’s frequency shift to return to 10% of its total frequency shift after the release of the target gas. Results showed that the response time increased from 200 s to 1800 s, and the recovery time increased from 230 s to 2400 s, respectively, when the ammonia concentration increased from 0.5 ppm to 50 ppm. The gap between these two values becomes more obvious, especially at a larger ammonia concentration. This is quite easily understood because the recovery time becomes slightly longer than the response time when the ammonia concentration is increased; thus, the difference between these two values turns to be dramatically increased at larger concentrations of ammonia.

Generally speaking, there are various reasons that could result in the changes of resonant frequency of the SAW device, including electrical loading (or acoustoelectric effect), mass loading, and elastic loading [26].

**Figure 8:**
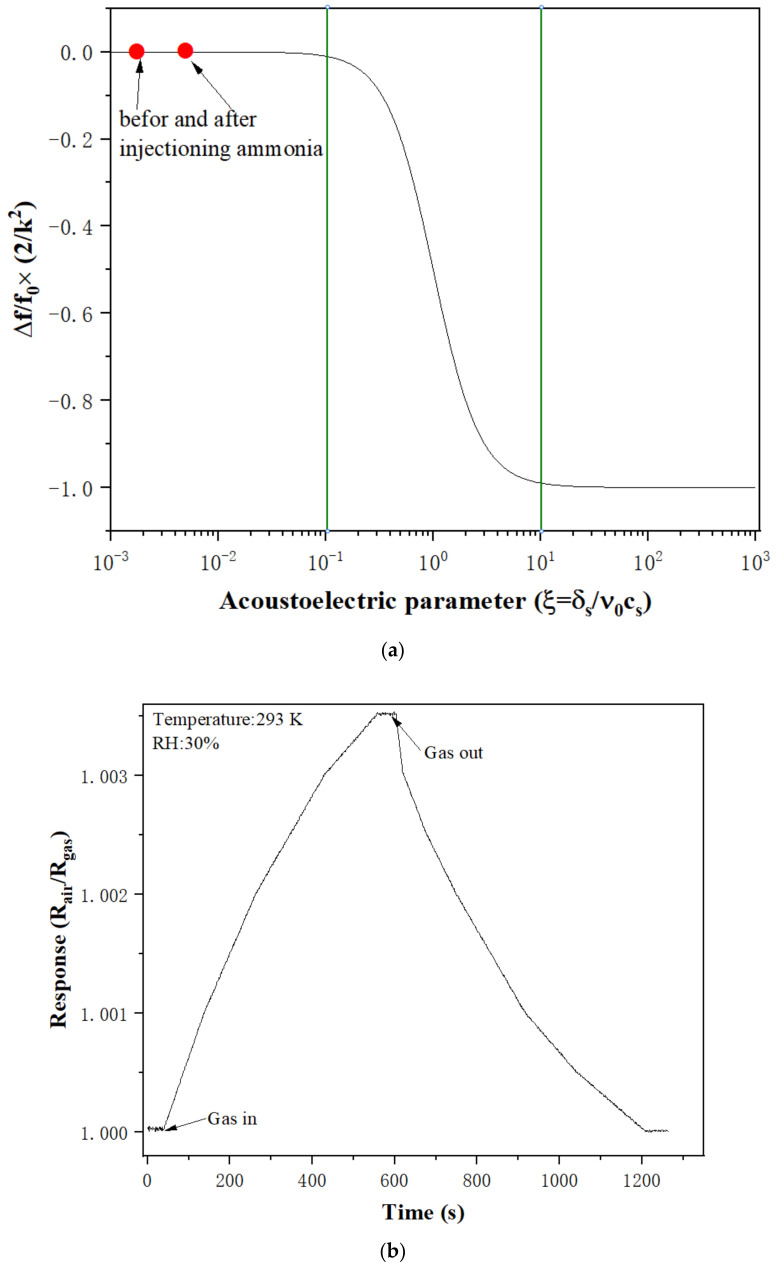
(**a**) Frequency changes of the PAA film SAW sensor versus acoustoelectric parameter ξ; (**b**) Resistance changes of PAA film before and after exposure to 20 ppm ammonia.

The relationship between frequency shift (Δ*f*) and electroacoustic effect can be expressed using the following equation [26,35]:

(1) $$\Delta f=-f_{0}\times \frac{K^{2}}{2}\times \left(\frac{1}{1+{\left(\frac{v_{0}c_{s}}{\sigma_{s}}\right)}^{2}}\right)$$

where *f*_0_ (~200 MHz), $K^{2}$ (0.11% for quartz), $v_{0}$ (3158 m/s for quartz), and $c_{s}$ (0.5 pF${\mathrm{cm}}^{-1}$) are the intrinsic resonant frequency of the SAW resonator, the electromechanical coupling factor, the unperturbed wave velocity for the SAW device, and the dielectric permittivity of the substrate and the environment, respectively. The measured sheet conductivity $\sigma_{s}$ of the PAA film in air and in 20 ppm ammonia in this study were $4.6\times 10^{-9}\;S/m$ and $\;4.6138\times 10^{-9}\;S/m,$ respectively. The corresponding acoustoelectric parameters ($\xi =\sigma_{s}/\upsilon_{0}C_{s}$) were $2.913\times 10^{-3}$ and $2.921\times 10^{-3}$, respectively, as shown in Figure 8a. According to Equation (1), at the initial condition with the PAA film exposed to air, the calculated value of $\Delta f_{1}/f_{0}$ was −4.99995 $\times \;10^{-4}$; however, when the device was exposed to 20 ppm ammonia, the value of $\Delta f_{2}/f_{0}$ changed to −5.00014 $\times \;10^{-4}$. Therefore, the calculated frequency shift $\Delta f=\Delta f_{2}-\Delta f_{1}$ = −(5.00014 $\times \;10^{-4}-4.99995\times 10^{-4}$) $\times f_{0}$ = −3.8 Hz. The calculated frequency shift is only −3.8 Hz for the PAA SAW sensor exposed to 20 ppm ammonia. Compared with the experimental data in this study, it can be concluded that electroacoustic effect is not the main mechanism leading to the frequency shift of the sensor.

A SAW electrode’s voltage and current were measured after the PAA film was exposed to 20 ppm of ammonia gas in order to further support this conclusion. Figure 8b shows the resistance responses $R=R_{air}/R_{gas}\;$ of the SAW device when it was exposed to 20 ppm ammonia gas, where $R_{air}$ and $R_{gas}$ are the resistances of the PAA film in the ambient air and in the ammonia/air mixture, respectively. As shown in Figure 8b, the resistance response R had no obvious changes (only from 1.000 to 1.0036).

The effect of mass loading on the frequency shift of sensors follows Equation (2) [26,35]

(2) $$\Delta f=\left(k_{1}+k_{2}\right)\times f_{0}^{2}\times \Delta \rho_{s}$$

where $k_{1}$ (−8.7 × 10^−8^ m^2^s kg^−1^), $k_{2}$ (−3.9 × 10^−9^ m^2^s kg^−1^), $f_{0}\;$ (~200 MHz), and $\Delta \rho_{s}$ are the material constants of the ST-cut quartz substrate, the intrinsic resonant frequency of SAW resonator, and the change of areal density of the sensing film under NH_3_ exposure, respectively. When the PAA film is exposed to ammonia gas, a negative frequency shift can be observed because the mass density of the PAA film is increased. Therefore, the experimentally obtained positive frequency shifts in this study reveal that mass loading should not be the primary mechanism in the frequency shifts of the PAA SAW sensor to ammonia.

Effect of elastic loading on the frequency shift of sensors follows Equation (3) [26,35]

(3) Δf=pΔE

where p and ΔE are a positive constant and the change of Young’s modulus of sensing film when exposed to the NH_3_ gas. The PAA has a large Young’s modulus, i.e., tens of GPa [36]. Due to the small size of ammonia molecules and the three-dimensional network structure of PAA, ammonia molecules are likely to be adsorbed by the PAA. The ammonia molecules filled in the PAA gap may increase the stiffness of the membrane, thus resulting in a positive frequency shift [26]. Therefore, we can confirm that elastic loading is the main factor affecting the frequency of PAA SAW ammonia sensor.

### 3.3. Selectivity and Stability of SAW Sensors

**Figure 9:**
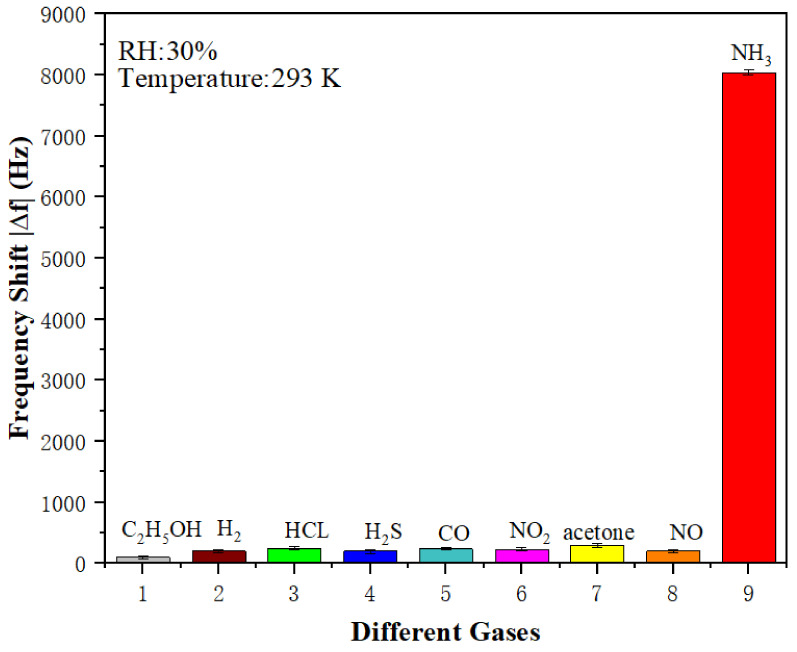
The absolute value of frequency shifts of PAA SAW ammonia sensor to different types of gases of 20 ppm.

Figure 9 shows the selectivity of the PAA SAW sensor to several major targeted gases with a fixed concentration of 20 ppm measured at room temperature of 293 K. As can be seen from Figure 9, the PAA SAW sensor had an obvious response to NH_3_ gas and its measured value of frequency shift was as high as 8000 Hz, whereas the values for the other gases were much lower. The results showed that the PAA SAW sensor had an excellent selectivity to ammonia.

**Figure 10:**
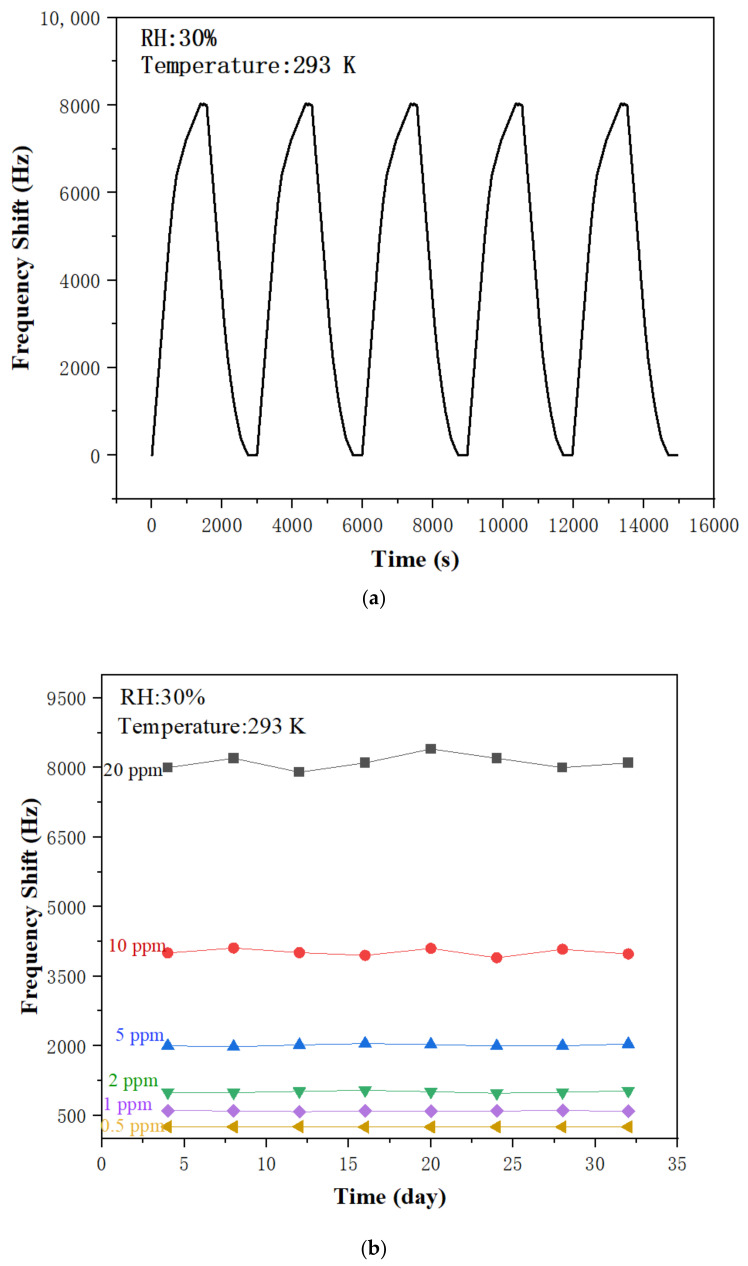
(**a**) Short-term repeatability of the PAA SAW sensor to 20 ppm ammonia; (**b**) long-term stability of the PAA SAW sensor to 20 ppm ammonia.

Figure 10a shows the dynamic responses of the sensor to five consecutive cycles of 20 ppm NH_3_ exposure. The measured frequency shifts are 8.04 kHz, 8.03 kHz, 8.03 kHz, 8.04 kHz, and 8.03 kHz, respectively, for these five cycles, which indicates that the PAA SAW sensor had good short-term repeatability. In order to study the long-term stability of the sensor, the sensor was tested by exposure to 0.5–20 ppm NH_3_ every 4 days for up to one month. As shown in Figure 10b, the sensor showed stable frequency shifts responses (less than 5%) to different concentrations of NH_3_ within a one-month period, which shows that it has good long-term stability.

### 3.4. Influences of Humidity and Temperature on Sensing Performance

**Figure 11:**
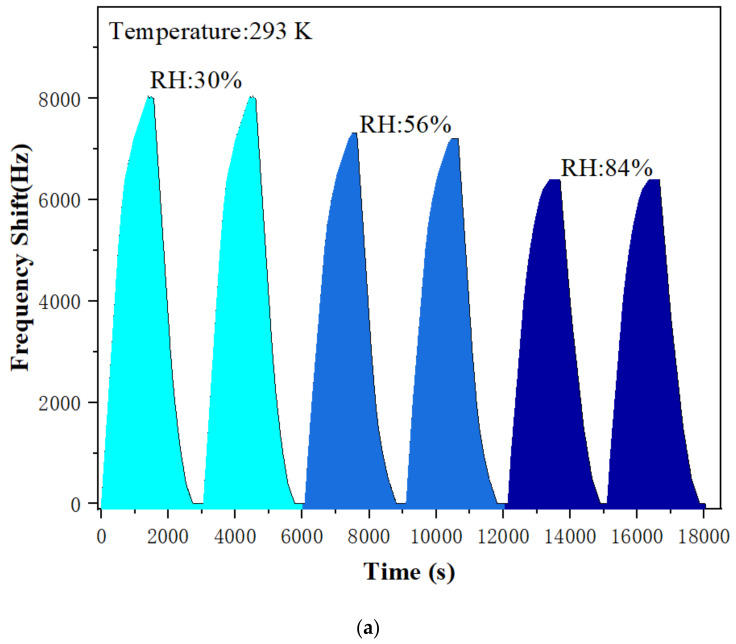

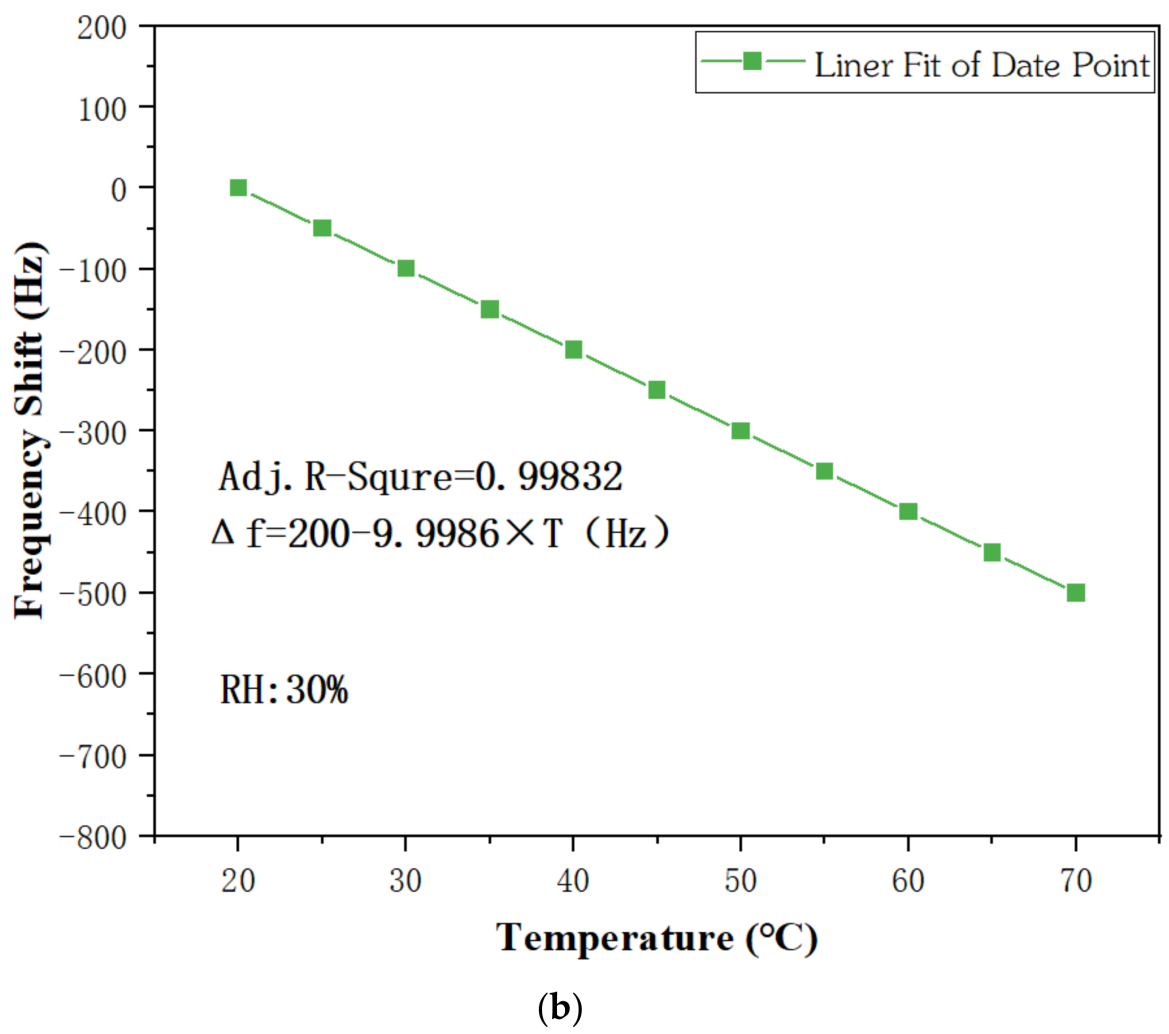
(**a**) Response–recovery curve of PAA SAW sensors to 20 ppm ammonia gas at room temperature of 293 K and different RH values; (**b**) the relationship between the resonant frequency shift of the PAA SAW sensor and temperature.

Humidity is one of the important influencing parameters of SAW gas sensor. Effects of different RH levels on the properties of PAA-sensitive membrane were studied and a humidifier was applied to control the relative humidity (RH) levels in the testing chamber. Figure 11a shows the sensing results of the PAA SAW sensor exposed to 20 ppm ammonia gas in different indoor environments with the RH values of 30%, 56%, and 84%, respectively. In the same concentration of ammonia, the frequency shift of the sensor decreases with the increase of RH. The reason is that with the increase of humidity, water molecules adsorbed by the hydroxyl groups in the PAA are increased, and these water molecules can become new absorption sites because of high solubility of ammonia in water. Thus, more ammonia molecules are absorbed and the mass loading effect is increased, which leads to a larger negative frequency shift. Therefore, the total positive frequency shift is decreased. However, we should address that the effects of RH levels were not significant, having a frequency shift of −1200 Hz when the RH level increased from 36% to 84%.

SAW sensors are also sensitive to temperature, and their dependences can be compared using the value of temperature coefficient of frequency (TCF). The TCF value of the device can be obtained to evaluate the thermal stability of the SAW device according to the following definitions:

(4) $$TCF=\frac{1}{\Delta T}\;\frac{\Delta f}{f_{0}}$$

where ΔT, Δf, and $f_{0}$ are the temperature change, the frequency change, and the center frequency of the SAW device, respectively. It is well known that the TCF of ST-Cut quartz crystal is near zero [37]. Figure 11b shows the variations of resonant frequencies of the PAA SAW sensor as a function of ambient temperature. When the temperature increased from 20 °C to 70 °C, the frequency shift increased up to 500 Hz. Since the center frequency of the SAW sensor is about 200 MHz, the calculated TCF was −0.049 ppm/°C, The results clearly showed that the slight change of temperature had little influences on the sensor’s performance and the PAA SAW ammonia sensor had an excellent thermal stability.

## 4. Conclusions

A highly sensitive and selective quartz-based SAW sensor with a sensing layer of PAA film was developed to detect ammonia at room temperature, which showed a good response to NH_3_ gas molecules. The sensor has a positive frequency shift of 225 Hz when exposed to 500 ppb NH_3_ gas. It had good selectivity for NH_3_ and good repeatability/stability. The oxygen-containing functional groups on the surfaces of PAA film make it sensitive and selective to ammonia gas molecules, even when tested at room temperature. The ammonia molecules adsorbed by the oxygen-containing functional groups of PAA (e.g., carboxyl groups, hydroxyl) increase the membrane’s stiffness, thus causing significantly elastic loading effect, which is identified as the primary mechanism leading to positive frequency shifts. Future work will be focused on a significant reduction of response and recovery times, and the synthesis of the nanostructured PAA composite membrane in order to enhance the performance and reduce the influence of humidity on the sensing performance.

## Figures and Tables

**Table 1:** Summary of the detection limits of various ammonia SAW sensors.

Sensitive Layer	Substrate	Detection Limit	References
**Co_3_O_4_/SiO_2_**	Quartz	1 ppm	[7]
**ZnO/SiO_2_ nanofilm**	Quartz	5 ppm	[10]
**Polyaniline film**	LiNbO_3_	25ppm	[17]
**L** **-glutamic acid hydrochloride nanofilm**	LiNbO_3_	5 ppm	[21]
**TiO_2_/SiO_2_ nanofilm**	Quartz	1 ppm	[26]
**ZnS**	Quartz	1 ppm	[27]
**SiO_2_/SiO_2_ nanofilm**	Quartz	3 ppm	[28]
**rGO/DPP2T-TT**	Quartz	0.5 ppm	[29]
**PAA film**	Quartz	0.5 ppm	this work
